# Functional Outcomes after Subtotal/Total Glossectomy with Microsurgical Reconstruction: A Multicenter Prospective Observational Study in Japan

**DOI:** 10.1245/s10434-025-17762-3

**Published:** 2025-07-12

**Authors:** Jun Araki, Keita Mori, Yoshichika Yasunaga, Masahiro Nakagawa, Tetsuro Onitsuka, Takashi Yurikusa, Yoshihiro Kimata, Minoru Sakuraba, Takuya Higashino, Ikuo Hyodo, Katsuhiro Ishida, Shimpei Miyamoto, Keisuke Takanari, Kaoru Sasaki, Masaki Arikawa, Hiroki Umezawa, Hideki Kadota, Hiroshi Matsumoto, Kentaro Tanaka, Miwako Fujii, Atsumori Hamahata, Tateki Kubo, Daisuke Yanagisawa, Koreyuki Kurosawa, Kohei Umekawa, Takuya Iida, Shunji Sarukawa, Hisashi Migita

**Affiliations:** 1 Division of Plastic and Reconstructive Surgery, Shizuoka Cancer Center Hospital, Shizuoka, Japan; 2 Department of Biostatistics, Shizuoka Cancer Center, Shizuoka, Japan; 3 Department of Plastic and Reconstructive Surgery, Shinshu University School of Medicine, Matsumoto, Japan; 4 Department of Plastic Reconstructive Surgery, Hamamatsu University School of Medicine, Shizuoka, Japan; 5 Division of Head and Neck Surgery, Shizuoka Cancer Center Hospital, Shizuoka, Japan; 6 Division of Dentistry and Oral Surgery, Shizuoka Cancer Center Hospital, Shizuoka, Japan; 7 Department of Plastic and Reconstructive Surgery, Okayama University Hospital, Okayama, Japan; 8 Department of Plastic and Reconstructive Surgery, Iwate Medical University, Morioka, Japan; 9 Department of Plastic and Reconstructive Surgery, National Cancer Center Hospital East, Kashiwa, Japan; 10 Department of Plastic and Reconstructive Surgery, University Hospital of Occupational and Environmental Health, Kitakyushu, Japan; 11 Department of Plastic and Reconstructive Surgery, Aichi Cancer Center Hospital, Aichi, Japan; 12 Department of Plastic and Reconstructive Surgery, The Jikei University School of Medicine, Tokyo, Japan; 13 Department of Plastic and Reconstructive Surgery, The University of Tokyo, Tokyo, Japan; 14 Division of Plastic and Reconstructive Surgery, National Cancer Center Hospital, Tokyo, Japan; 15 Department of Plastic and Reconstructive Surgery, Faculty of Medicine, University of Tsukuba, Tsukuba, Japan; 16 Department of Plastic, Reconstructive, and Aesthetic Surgery, Nippon Medical School Hospital, Tokyo, Japan; 17 Department of Plastic and Reconstructive Surgery, Kyushu University Hospital, Fukuoka, Japan; 18 Department of Reconstructive Plastic Surgery, Graduate School of Medical and Dental Sciences, Institute of Science Tokyo, Tokyo, Japan; 19 Department of Plastic and Reconstructive Surgery, Tokyo Metropolitan Cancer and Infectious Diseases Center Komagome Hospital, Tokyo, Japan; 20 Department of Plastic and Reconstructive Surgery, Saitama Cancer Center, Saitama, Japan; 21 Department of Plastic Surgery, The University of Osaka Graduate School of Medicine, Suita, Japan; 22 Department of Plastic and Reconstructive Surgery, Tohoku University Graduate School of Medicine, Sendai, Japan; 23 Department of Plastic and Reconstructive Surgery, Dokkyo Medical University School of Medicine, Tochigi, Japan; 24 Department of Plastic and Reconstructive Surgery, Saitama Medical University International Medical Center, Saitama, Japan; 25 Department of Plastic and Reconstructive Surgery and Maxillofacial Surgery, Kurume University School of Medicine, Kurume, Japan

**Keywords:** Subtotal/total tongue reconstruction, Feeding tube dependence, Oral intake level, Speech function, Microsurgical reconstruction

## Abstract

**Background:**

This prospective observational multicenter study aimed to provide evidence-based data on the risk factors for feeding tube dependence, oral intake level, and speech function after tongue reconstruction.

**Patients and Methods:**

This study was conducted by the Oral Pharyngeal Esophageal Operation and Reconstruction Analytical group across 21 Japanese institutions. Patients with oral tongue squamous cell carcinoma who underwent microsurgical reconstruction following subtotal/total glossectomy were included. Functional evaluations were performed 1 year postoperatively. The primary endpoint was postoperative feeding tube dependence. The other outcome variables were oral intake level and speech function at the time of evaluation.

**Results:**

Overall, 189 patients were enrolled, of whom 121 (64.0%) were followed up for 1 year after surgery and were eligible for the final analysis. The overall rate of feeding tube dependence was 12.4% (*n* = 15) at the primary endpoint. Univariate analysis revealed that tongue defect type, laryngeal suspension, and postoperative chemotherapy were associated with feeding tube dependence. The oral intake level was affected by age at surgery, laryngeal suspension, and postoperative chemoradiation. Speech function was affected by age at surgery, American Society of Anesthesiologists physical status (class 2), medical comorbidities of hypertension and cardiac dysrhythmia, primary tumor stage (T4), neck dissection, reconstructive procedure, laryngeal suspension, and postoperative radiation.

**Conclusions:**

This study provides useful data for estimating individual risk factors associated with feeding tube dependence, oral intake level, and speech function before tongue reconstruction. These results can help surgeons and patients make informed decisions and optimize the functional outcomes of tongue reconstruction.

Oral tongue squamous cell carcinoma (OTSCC) is an orphan malignant disease (ORPHA: 457252, www.orpha.net), and its incidence is increasing globally.^1^ Oral cavity squamous cell carcinoma, including OTSCC, is a head and neck tumor characterized by a firm infiltrative neoplasm with squamous differentiation arising from the mucosal epithelium. These tumors are most commonly located in the tongue, floor of the mouth, or gingiva and may also infiltrate or affect the buccal mucosa or other areas of the oral cavity. Tumor location is dependent on the prevailing risk factors, including smoking and alcohol consumption. Patients present with various white, erythematous, mixed, nodular, or ulcerated lesions that may cause discomfort, pain, or reduced tongue mobility. The tumor is aggressive, showing a tendency for local invasion and early lymph node metastasis.

OTSCC is staged in accordance with the tumor-node-metastasis (TNM) classification of malignant tumors. Resectable tumors are treated surgically. Chemotherapy and/or radiotherapy is considered for patients with unresectable tumors. An appropriate surgical procedure is selected for resectable tumors on the basis of patient age, general health status, and occupation. Some patients experience permanent disabilities, including dysphagia and dysarthria, as a result of glossectomy. If half or more of the tongue is resected (hemiglossectomy or subtotal or total glossectomy), immediate reconstructive surgery with a pedicled or free flap is required. Diverse reconstructive procedures are employed, with differences based on surgeon preferences and individual institution preferences. There is no consensus on postoperative outcomes, although several small single-center studies have been published on this topic.^2–11^ Recently, our group reported a risk model for predicting feeding tube dependence after tongue reconstruction in a retrospective multicenter study in Japan.^12^ However, retrospective studies are generally considered to be of inferior quality because exposures are investigated after measuring outcomes, which can lead to selection bias.

To the best of our knowledge, this is the first prospective observational multicenter study on functional outcomes after tongue reconstruction. We prospectively examined the incidence of tube feeding dependence again. Moreover, we conducted an exploratory assessment regarding the food form of oral intake and outcomes related to speech function. This analysis was statistically powered by logistic regression analyses of 121 cases from 21 institutions.

## Patients and Methods

### Setting and Study Design

This prospective multicenter study was conducted by the Oral Pharyngeal Esophageal Operation and Reconstruction Analytical (OPERA) study group across 21 cancer centers and university hospitals in Japan. The participating institutions and investigators are listed in eTable 1. All patients diagnosed with OTSCC who underwent tongue reconstruction after subtotal or total glossectomy between September 2017 and August 2020 were included. The inclusion criteria were a local diagnosis of OTSCC at the initial histopathological examination of the primary tumor and tongue reconstruction via free flap transfer. The exclusion criteria were distant metastases, simultaneous double cancer, laryngectomy, segmental mandibulectomy, and recurrence during the observation period. The study protocol was approved by the Ethics Committee of the Shizuoka Cancer Center Hospital (approval no.: T28-65-29-1-2) and subsequently by all participating institutions. All procedures, including obtaining informed consent, were conducted in accordance with the ethical standards of the Committee on Human Experimentation of the participating institutions.

### Data Acquisition

The clinical data (patient background, intraoperative and postoperative findings, and functional outcomes at 1 year after surgery) of the candidates were retrieved using a uniform case report form from the individual institutions. In case of discrepancies, the original patient health records were thoroughly reviewed. Patient data included age, sex, American Society of Anesthesiologists (ASA) physical status classification, body mass index, blood albumin level, blood hemoglobin level, tobacco use, alcohol consumption, medical comorbidities (hypertension, diabetes, cardiac dysrhythmia, peripheral vascular diseases, cranial neuropathy, neuromuscular disorders, and psychiatric disorders), and preoperative therapies (radiation, chemotherapy, and surgery). Perioperative data included TNM classification, tongue defects (types 1–5), resection beyond the tongue (including the buccal mucosa, lateral wall of the oropharynx, and/or the marginal mandible), neck dissection, reconstructive procedures (rectus abdominis myocutaneous [RAM], anterolateral thigh [ALT], forearm, or groin) flap, operation time, blood loss, tracheotomy, laryngeal suspension, cricopharyngeal myotomy, postoperative complications, additional resection (as a secondary surgery), reoperation for flap-related complications, postoperative therapies (radiation, chemotherapy, or chemoradiation), rehabilitation, oral care, tongue shape (defined by appearance as protuberant, flat, or depressed), and functional teeth.

Laryngeal suspension is indicated to mitigate aspiration risk and enhance swallowing function, particularly following extensive tongue resections that compromise the suprahyoid musculature. By elevating the larynx, this procedure aids in restoring airway protection mechanisms. Cricopharyngeal myotomy is considered when patients exhibit oropharyngeal dysphagia due to impaired relaxation of the upper esophageal sphincter, despite adequate laryngeal elevation and pharyngeal pressure. This condition may arise after tongue reconstruction, especially when pharyngeal propulsion is compromised.

**Fig. 1:**
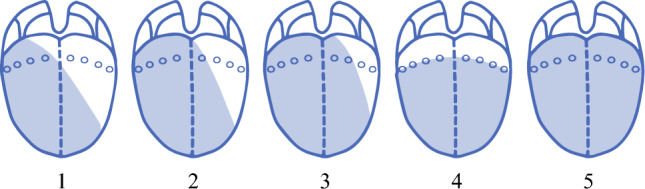
Extent of glossectomy: type 1, subtotal oral glossectomy with partial resection of the base of the tongue; type 2, subtotal oral glossectomy with hemiresection of the tongue base; type 3, subtotal glossectomy (subtotal resection of the whole tongue); type 4, total oral glossectomy (total resection of the oral tongue); and type 5, total glossectomy (total resection of the whole tongue)

The extent of glossectomy was classified into five types (Fig. 1) as follows: type 1, subtotal oral glossectomy with partial resection of the base of the tongue; type 2, subtotal oral glossectomy with hemiresection of the tongue base; type 3, subtotal glossectomy (subtotal resection of the whole tongue); type 4, total oral glossectomy (total resection of the oral tongue); and type 5, total glossectomy (total resection of the whole tongue).

### Outcomes

Functional evaluations were performed 1 year after surgery by outpatient clinicians. The primary endpoint was complete dependence on a postoperative feeding tube. The secondary endpoints included oral intake level, evaluated using the Method of intake, Time, and Food (MTF) scoring system,^13^ in patients who were independent of the feeding tube, as well as speech intelligibility, evaluated using the Hirose scoring system.^14^

### Statistical Analyses

Statistical analyses were conducted using R version 4.3.2 (R Foundation for Statistical Computing, Vienna, Austria). Median, maximum, and minimum values were calculated for continuous data. The frequency of each category was calculated for categorical data. Univariate logistic regression models were constructed to identify associations with postoperative feeding tube dependence. As this was an exploratory study, multiple testing corrections were not deemed appropriate. *P*-values of < 0.05 were considered statistically significant.

## Results

### Recruitment and Study Population

**Fig. 2:**
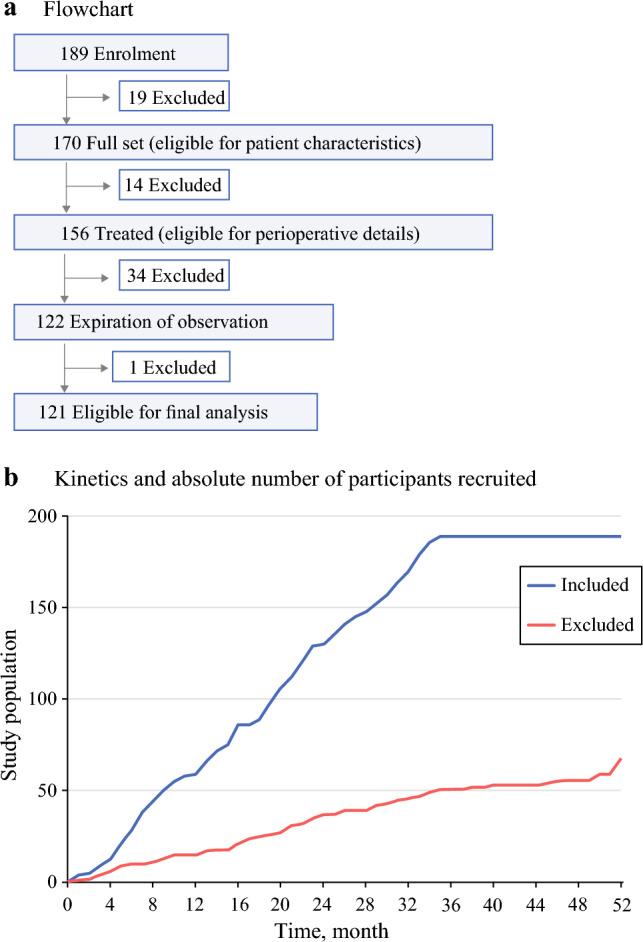
Flowchart and absolute number of participants recruited; of the 189 patients enrolled, 121 (64.0%) were eligible for the final analysis **A**; the study populations included and excluded in this study are shown in a time series graph **B**

This study aimed to include 200 patients, sufficient for multivariate analyses; however, enrollment did not proceed as smoothly as expected. Of the 189 patients enrolled, 19 withdrew due to ineligibility. A total of 170 (89.9%) patients were selected on the basis of their characteristics, and 14 withdrew after undergoing miniaturized or modified resection. In total, 156 (82.5%) patients received treatment; 34 could not be evaluated 1 year after surgery: 24 patients died during the observation period, and 8 patients chose to discontinue the study. Feeding tube dependence, tracheostomy dependence, and cause of death are relevant to the interpretation of the remaining data. One patient could not be evaluated due to the coronavirus disease pandemic. Overall, 121 (64.0%) patients were evaluated 1 year after surgery and were eligible for the final analysis (Fig. 2A). In total, 189 patients were enrolled. Perioperative details were presented for 156 treated patients, and final analyses were conducted on 121 patients who could be followed until the study endpoints 1 year after surgery. The study populations included and excluded from this study are shown in a time series graph (Fig. 2B).

### Clinical Characteristics

Patient characteristics are presented in eTable 2. The median age at surgery was 64 (range 21–88) years, and the study population included 125 (73.5%) male patients and 45 (26.5%) female patients. ASA physical status, a commonly used perioperative assessment parameter,^15^ was used to evaluate the patients’ general condition. Overall, 115 (67.6%) patients were classified as class 1, 50 (29.4%) as class 2, and 5 (2.9%) as class 3; no patients were classified into classes 4–6. The median body mass index was 22.0 (range, 15.5–32.0) kg/m^2^, and the median serum albumin and hemoglobin levels were 4.1 (1.9–5.0) g/dL and 13.5 (8.4–17.4) g/dL, respectively. A total of 98 (57.6%) patients had a smoking history (median Brinkman index, 600 [15–1800]). A total of 130 (76.5%) patients had a history of alcohol consumption. As for medical comorbidities, 61 (35.9%) patients had hypertension, 32 (18.8%) had diabetes, 18 (10.6%) had cardiac dysrhythmia, 2 (1.2%) had peripheral vascular disease, 13 (7.6%) had cerebral disease, 2 (1.2%) had neuromuscular disorders, and 5 (2.9%) had psychiatric disorders. Regarding preoperative therapies, 1 (0.6%) patient underwent radiation (dose: 40 Gy), 20 (11.8%) underwent chemotherapy, and 14 (8.2%) underwent surgery.

The perioperative details of the 156 treated cases are presented in eTable 3. Tumor staging was performed according to the TNM classification (Union for International Cancer Control, 8th edition).^16,17^ The most common type of tongue defect was type 1 (62 [39.7%] patients), followed by type 2 (33 [21.2%] patients), type 3 (33 [16.4%] patients), type 4 (20 [12.8%] patients), and type 5 (8 [5.1%] patients). Resection beyond the tongue was performed in 58 (37.2%) patients. Specifically, resections of the buccal mucosa, lateral wall of the oropharynx, and marginal mandible were performed in 4 (2.6%), 51 (32.7%), and 17 (10.9%) patients, respectively, with some patients undergoing multiple resections. All patients underwent neck dissection: 53 (34.0%) unilaterally and 103 (66.0%) bilaterally. The most common tongue reconstruction procedure was the RAM flap (90 [57.7%] patients), followed by the ALT flap (64 [41.0%] patients), forearm flap (1 [0.6%] patient), and groin flap (1 [0.6%] patient). The median operation time and blood loss were 558 (354–1250) min and 324.5 (33–1620) mL, respectively. Tracheotomy, laryngeal suspension, and cricopharyngeal myotomy were performed in all patients (156 [100%]), 71 (45.5%), and 17 (10.9%), respectively. Additional resections and reoperations for flap-related complications were performed in zero (0%) and five (3.2%) patients at the time of discharge, respectively. Postoperative therapy was administered to 81 (51.9%) patients, including radiation alone in 17 (10.9%), chemotherapy alone in 5 (3.2%), and chemoradiation in 59 (37.8%). Adjuvant chemotherapy alone is not routinely recommended following surgery for tongue cancer. Its use was considered only in specific patient circumstances where radiotherapy was contraindicated. Rehabilitation for dysphagia and oral care was provided to 155 (99.4%) patients. The tongue shape was protuberant in 141 (90.4%) patients, flat in 12 (7.7%), and depressed in 3 (1.9%) at the time of discharge.

### Predictive Factors of Feeding Tube Dependence

In total, 189 patients were enrolled, 121 (64.0%) of whom were eligible for the final analysis. The overall rate of feeding tube dependence at the primary endpoint was 12.4% (*n* = 15). Univariate analysis revealed that tongue defect type 3 (*P* = 0.0168), laryngeal suspension (*P* = 0.0344), and postoperative chemotherapy (*P* = 0.0149) and chemoradiation (*P* = 0.0333) were positively associated with feeding tube dependence. *P*-values of < 0.05 were considered statistically significant and are marked with an asterisk in Table 1.

**Table 1:** Prognostic factors associated with postoperative feeding tube dependence in univariate analysis (*n* = 121)

Variable	Feeding tube dependence			
	Yes (%)	No (%)	OR (95% CI)	*P*
Age at surgery, years				
Median	69	60	0.98 (0.94–1.02)	0.239
Range	21–79	22–83		
Sex				
Male (*n* = 89)	11 (12.4%)	78 (87.6%)	1	
Female (*n* = 32)	4 (12.5%)	28 (87.5%)	1.01 (0.30–3.44)	0.984
ASA physical status classification				
1 (*n* = 85)	7 (8.2%)	78 (91.8%)	1	
2 (*n* = 33)	7 (21.2%)	26 (78.8%)	3.00 (0.96–9.36)	0.058
3 (*n* = 3)	1 (33.3%)	2 (66.7%)	5.57 (0.45–69.4)	0.182
4 (*n* = 0)	0	0	NA	NA
5 (*n* = 0)	0	0	NA	NA
6 (*n* = 0)	0	0	NA	NA
Body mass index, kg/m^2^				
Median	22	21.9	1.07 (0.90–1.27)	0.431
Range	15.8–25.8	15.5–31.6		
Albumin, g/dL				
Median	4.2	4.1	1.15 (0.30–4.56)	0.84
Range	3.3–4.8	2.9–5.0		
Hemoglobin, g/dL				
Median	13.5	13.7	1.01 (0.71–1.44)	0.962
Range	10.5–16.3	9.0–17.4		
Tobacco use				
Never (*n* = 36)	5 (13.9%)	31 (86.1%)	1	
Ever (*n* = 49)	6 (12.2%)	43 (87.8%)	0.87 (0.24–3.09)	0.824
Current (*n* = 36)	4 (11.1%)	32 (88.9%)	0.78 (0.19–3.16)	0.722
Alcohol consumption				
Never (*n* = 32)	6 (18.75%)	26 (81.25%)	1	
Ever (*n* = 14)	1 (7.1%)	13 (92.9%)	0.33 (0.04–3.07)	0.332
Sometimes (*n* = 29)	3 (10.3%)	26 (89.7%)	0.50 (0.11–2.22)	0.362
Almost every day (*n* = 46)	5 (10.9%)	41 (89.1%)	0.53 (0.15–1.91)	0.331
Medical comorbidities				
Hypertension				
Yes (*n* = 44)	7 (15.9%)	37 (84.1%)	1.63 (0.55–4.85)	0.379
No (*n* = 77)	8 (10.4%)	69 (89.6%)	1	
Diabetes				
Yes (*n* = 19)	3 (15.8%)	16 (84.2%)	1.41 (0.36–5.55)	0.626
No (*n* = 102)	12 (11.8%)	90 (88.2%)	1	
Cardiac dysrhythmia				
Yes (*n* = 10)	3 (30.0%)	7 (70.0%)	3.54 (0.81–15.5)	0.094
No (*n* = 111)	12 (10.8%)	99 (89.2%)	1	
Peripheral vascular disease				
Yes (*n* = 1)	0 (0%)	1 (100%)	NA	NA
No (*n* = 120)	15 (12.5%)	105 (87.5%)	NA	
Cerebral disease				
Yes (*n* = 7)	2 (28.6%)	5 (71.4%)	3.11 (0.55–17.7)	0.201
No (*n* = 114)	13 (11.4%)	101 (88.6%)	1	
Neuromuscular disorder				
Yes (*n* = 1)	1 (100%)	0 (0%)	NA	NA
No (*n* = 120)	14 (11.7%)	106 (88.3%)	NA	
Psychiatric disorder				
Yes (*n* = 4)	0 (0%)	4 (100%)	NA	NA
No (*n* = 117)	15 (12.8%)	102 (87.2%)	NA	
Preoperative therapy				
Radiation				
Yes (*n* = 1)	0 (0%)	1 (100%)	NA	NA
Dose, Gy	–	40		
No (*n* = 120)	15 (12.5%)	105 (87.5%)	NA	
Chemotherapy				
Yes (*n* = 12)	1 (8.3%)	11 (91.7%)	0.62 (0.07–5.15)	0.656
No (*n* = 109)	14 (12.8%)	95 (87.2%)	1	
Surgery				
Yes (*n* = 11)	0 (0%)	11 (100%)	NA	NA
No (*n* = 110)	15 (13.6%)	95 (86.4%)	NA	
TNM classification				
Primary tumor (T)				
T0 (*n* = 0)	0	0	NA	NA
T1 (*n* = 1)	0 (0%)	1 (100%)	NA	NA
T2 (*n* = 9)	0 (0%)	9 (100%)	NA	NA
T3 (*n* = 67)	8 (11.9%)	59 (88.1%)	1	
T4 (*n* = 42)	7 (16.7%)	35 (83.3%)	1.48 (0.49–4.42)	0.488
TX (*n* = 0)	0	0	NA	NA
Recurrence (*n* = 2)	0 (0%)	2 (100%)	NA	NA
Regional lymph nodes (N)				
N0 (*n* = 56)	7 (12.5%)	49 (87.5%)	1	
N1 (*n* = 16)	3 (18.75%)	13 (81.25%)	1.62 (0.37–7.13)	0.527
N2 (*n* = 41)	5 (12.2%)	36 (87.8%)	0.97 (0.29–3.31)	0.964
N3 (*n* = 6)	0 (0%)	6 (100%)	NA	NA
NX (*n* = 0)	0	0	NA	NA
Missing (*n* = 2)	0	2	NA	NA
Tongue defect type				
1: (*n* = 55)	2 (3.6%)	53 (96.4%)	1	
2: (*n* = 23)	4 (17.4%)	19 (82.6%)	5.58 (0.94–33.0)	0.058
3: (*n* = 21)	5 (23.8%)	16 (76.2%)	8.28 (1.46–46.8)	0.017*
4: (*n* = 16)	3 (18.75%)	13 (81.25%)	6.12 (0.93–40.5)	0.06
5: (*n* = 6)	1 (16.7%)	5 (83.3%)	5.30 (0.41–69.2)	0.203
Resection beyond the tongue				
Yes (*n* = 44)	8 (18.2%)	36 (81.8%)	0.45 (0.15–1.34)	0.152
No (*n* = 77)	7 (9.1%)	70 (90.1%)	1	
Neck dissection				
None (*n* = 0)	0	0	NA	NA
One side (*n* = 46)	4 (8.7%)	42 (91.3%)	1	
Both sides (*n* = 75)	11 (14.7%)	64 (85.3%)	1.81 (0.54–6.04)	0.338
Reconstructive procedure				
RAM (*n* = 67)	11 (16.4%)	56 (83.6%)	1	
ALT (*n* = 52)	4 (7.7%)	48 (92.3%)	0.42 (0.13–1.42)	0.164
Others (*n* = 2)	0 (0%)	2 (100%)	NA	NA
Operation time, min				
Median	559	535	1.00 (1.00–1.00)	0.834
Range	434–1183	354–1250		
Bleeding, mL				
Median	500	317	1.00 (1.00–1.00)	0.089
Range	155–1029	33–1380		
Tracheotomy				
Yes (*n* = 121)	15 (12.4%)	106 (87.6%)	NA	NA
No (*n* = 0)	0	0	NA	
Laryngeal suspension				
Yes (*n* = 49)	10 (20.4%)	39 (79.6%)	3.44 (1.10–10.8)	0.034*
No (*n* = 72)	5 (6.9%)	67 (93.1%)	1	
Cricopharyngeal myotomy				
Yes (*n* = 15)	2 (13.3%)	13 (86.7%)	1.10 (0.22–5.44)	0.906
No (*n* = 106)	15 (14.2%)	91 (85.8%)	1	
Additional resection				
Yes (*n* = 11)	1 (9.1%)	10 (90.9%)	1.46 (0.17–12.3)	0.729
No (*n* = 110)	14 (12.7%)	96 (87.3%)	1	
Reoperation for flap-related complications				
Yes (*n* = 5)	1 (20.0%)	4 (80.0%)	1.82 (0.19–17.5)	0.603
No (*n* = 116)	14 (12.1%)	102 (87.9%)	1	
Postoperative therapy				
No (*n* = 54)	3 (5.6%)	51 (94.4%)	1	
Radiation alone (*n* = 14)	0 (0%)	14 (100%)	NA	NA
Chemotherapy alone (*n* = 4)	2 (50%)	2 (50%)	17 (1.74–166)	0.015*
Chemoradiation (*n* = 49)	10 (20.4%)	39 (79.6%)	4.36 (1.12–16.9)	0.033*
Rehabilitation				
Yes (*n* = 121)	15 (12.4%)	106 (87.6%)	NA	
No (*n* = 0)	0	0	NA	NA
Oral care				
Yes (*n* = 120)	15 (12.5%)	105 (87.5%)	NA	
No (*n* = 1)	0 (0%)	1 (100%)	NA	NA
Shape of the tongue				
Protuberant (*n* = 97)	11 (11.3%)	86 (88.7%)	1	
Flat (*n* = 20)	4 (20.0%)	16 (80.0%)	1.96 (0.55–6.91)	0.298
Depressed (*n* = 4)	0 (0%)	4 (100%)	NA	NA

*ALT* Anterolateral thigh, *ASA* American Society of Anesthesiologists, *CI* Confidence interval, *OR* Odds ratio, *RAM* Rectus abdominis myocutaneous, *TNM* Tumor-node-metastasis

^*^P-values of < 0.05 were considered statistically significant and are indicated with an asterisk.

### Prognostic Factors Associated with Oral Intake Level in Univariate Analysis

Feeding tube-independent patients (*n* = 106) were eligible for this analysis. Oral intake was assessed as limited (M3 or M4 on the MTF scoring system) or non-limited (M5 on the MTF scoring system). Univariate analysis revealed that oral intake level was negatively influenced by age at surgery (*P* = 0.0071), laryngeal suspension (*P* = 0.0035), and postoperative chemoradiation (*P* = 0.0207). *P*-values of < 0.05 were considered statistically significant and are marked with an asterisk in Table 2.

**Table 2:** Prognostic factors associated with oral intake level in univariate analysis (*n* = 106)

Variable	Oral intake level			
	Limited (%)	Non-limited (%)	OR (95% CI)	*P*
Age at surgery, years				
Median	65.5	56.5	1.04 (1.01–1.07)	0.007*
Range	26–83	22–82		
Sex				
Male (*n* = 78)	38 (48.7%)	40 (51.3%)	1	
Female (*n* = 28)	18 (64.3%)	10 (35.7%)	1.90 (0.78–4.62)	0.16
ASA physical status classification				
1 (*n* = 78)	39 (50.0%)	39 (50.0%)	1	
2 (*n* = 26)	15 (57.7%)	11 (42.3%)	1.36 (0.56–3.34)	0.497
3 (*n* = 2)	2 (100%)	0 (0%)	NA	NA
4 (*n* = 0)	0	0	NA	NA
5 (*n* = 0)	0	0	NA	NA
6 (*n* = 0)	0	0	NA	NA
Body mass index, kg/m^2^				
Median	22.2	21.7	0.98 (0.87–1.10)	0.692
Range	15.5–29.4	15.7–31.6		
Albumin, g/dL				
Median	4	4.2	0.40 (0.14–1.12)	0.082
Range	3.1–5.0	2.9–4.9		
Hemoglobin, g/dL				
Median	13.4	13.9	0.77 (0.58–1.01)	0.061
Range	9.0–15.2	10.4–17.4		
Tobacco use				
Never (*n* = 31)	15 (48.4%)	16 (51.6%)	1	
Ever (*n* = 43)	27 (62.8%)	16 (37.2%)	1.80 (0.71–4.60)	0.219
Current (*n* = 32)	14 (43.75%)	18 (56.25%)	0.83 (0.31–2.24)	0.712
Alcohol consumption				
Never (*n* = 26)	12 (46.2%)	14 (53.8%)	1	
Ever (*n* = 13)	9 (69.2%)	4 (30.8%)	2.63 (0.64–10.7)	0.179
Sometime (*n* = 26)	13 (50.0%)	13 (50.0%)	1.17 (0.39–3.47)	0.781
Almost every day (*n* = 41)	22 (53.7%)	19 (46.3%)	1.35 (0.50–3.62)	0.55
Medical comorbidities				
Hypertension				
Yes (*n* = 37)	20 (54.1%)	17 (45.9%)	1.08 (0.48–2.40)	0.853
No (*n* = 69)	36 (52.2%)	33 (47.8%)	1	
Diabetes				
Yes (*n* = 16)	8 (50.0%)	8 (50.0%)	0.88 (0.32–2.54)	0.806
No (*n* = 90)	48 (53.3%)	42 (46.7%)	1	
Cardiac dysrhythmia				
Yes (*n* = 7)	3 (42.9%)	4 (57.1%)	0.65 (0.14–3.06)	0.587
No (*n* = 99)	53 (53.5%)	46 (46.5%)	1	
Peripheral vascular disease				
Yes (*n* = 1)	0 (0%)	1 (100%)	NA	NA
No (*n* = 105)	56 (53.3%)	49 (46.7%)	NA	
Cerebral disease				
Yes (*n* = 5)	4 (80.0%)	1 (20.0%)	3.77 (0.41–34.9)	0.243
No (*n* = 101)	52 (51.5%)	49 (48.5%)	1	
Neuromuscular disorder				
Yes (*n* = 0)	0	0	NA	NA
No (*n* = 106)	56 (52.8%)	50 (47.2%)	NA	
Psychiatric disorder				
Yes (*n* = 4)	3 (75.0%)	1 (25.0%)	2.77 (0.28–27.6)	0.384
No (*n* = 102)	53 (52.0%)	49 (48.0%)	1	
Preoperative therapy				
Radiation				
Yes (*n* = 1)	0 (0%)	1 (100%)	NA	NA
Dose, Gy	–	40		
No (*n* = 105)	56 (53.3%)	49 (46.7%)	NA	
Chemotherapy				
Yes (*n* = 11)	6 (54.5%)	5 (45.5%)	1.08 (0.31–3.78)	0.904
No (*n* = 95)	50 (52.6%)	45 (47.4%)	1	
Surgery				
Yes (*n* = 11)	7 (63.6%)	4 (36.4%)	1.64 (0.45–5.98)	0.452
No (*n* = 95)	49 (51.6%)	46 (48.4%)	1	
TNM classification				
Primary tumor (T)				
T0 (*n* = 0)	0	0	NA	NA
T1 (*n* = 1)	0 (0%)	1 (100%)	NA	NA
T2 (*n* = 9)	5 (55.6%)	4 (44.4%)	1.30 (0.32–5.30)	0.721
T3 (*n* = 59)	29 (49.2%)	30 (50.8%)	1	
T4 (*n* = 35)	21 (60.0%)	14 (40.0%)	1.55 (0.67–3.62)	0.309
TX (*n* = 0)	0	0	NA	NA
Recurrence (*n* = 2)	1 (50%)	1 (50%)	1.03 (0.06–17.3)	0.981
Regional lymph nodes (N)				
N0 (*n* = 49)	23 (46.9%)	26 (53.1%)	1	
N1 (*n* = 13)	9 (69.2%)	4 (30.8%)	2.54 (0.69–9.38)	0.261
N2 (*n* = 36)	20 (55.6%)	16 (44.4%)	1.41 (0.60–3.35)	0.433
N3 (*n* = 6)	3 (50%)	3 (50%)	1.13 (0.21–6.16)	0.887
NX (*n* = 0)	0	0	NA	NA
Missing (*n* = 2)	1	1	NA	NA
Tongue defect type				
1: (*n* = 53)	25 (47.2%)	28 (52.8%)	1	
2: (*n* = 19)	10 (52.6%)	9 (47.4%)	1.24 (0.44–3.56)	0.683
3: (*n* = 16)	10 (62.5%)	6 (37.5%)	1.87 (0.59–5.88)	0.286
4: (*n* = 13)	8 (61.5%)	5 (38.5%)	1.80 (0.52–6.20)	0.357
5: (*n* = 5)	3 (60.0%)	2 (40.0%)	1.68 (0.26–10.9)	0.586
Resection beyond the tongue				
Yes (*n* = 36)	17 (47.2%)	19 (52.8%)	0.71 (0.32–1.59)	0.408
No (*n* = 70)	39 (55.7%)	31 (44.3%)	1	
Neck dissection				
None (*n* = 0)	0	0	NA	NA
One side (*n* = 42)	19 (45.2%)	23 (54.8%)	1	
Both sides (*n* = 64)	37 (57.8%)	27 (42.2%)	1.66 (0.76–3.64)	0.206
Reconstructive procedure				
RAM (*n* = 56)	33 (58.9%)	23 (41.0%)	1	
ALT (*n* = 48)	22 (45.8%)	26 (54.2%)	0.59 (0.27–1.28)	0.184
Forearm (*n* = 1)	1 (100%)	0 (0%)	NA	NA
Groin (*n* = 1)	0 (0%)	1 (100%)	NA	NA
Operation time, min				
Median	532.5	552	1.00 (1.00–1.00)	0.262
Range	354–916	385–1250		
Bleeding, mL				
Median	300	333	1.00 (1.00–1.00)	0.974
Range	50–1010	33–1380		
Tracheotomy				
Yes (*n* = 106)	56 (52.8%)	50 (47.2%)	NA	NA
No (*n* = 0)	0	0	NA	
Laryngeal suspension				
Yes (*n* = 39)	28 (71.8%)	11 (28.2%)	3.55 (1.52–8.29)	0.004*
No (*n* = 67)	28 (41.8%)	39 (58.2%)	1	
Cricopharyngeal myotomy				
Yes (*n* = 13)	9 (69.2%)	4 (30.8%)	2.20 (0.63–7.66)	0.214
No (*n* = 93)	47 (50.5%)	46 (49.5%)	1	
Additional resection				
Yes (*n* = 10)	8 (80.0%)	2 (20.0%)	4.00 (0.81–19.8)	0.09
No (*n* = 96)	48 (50.0%)	48 (50.0%)	1	
Reoperation for flap-related complications				
Yes (*n* = 4)	3 (75.0%)	1 (25.0%)	2.77 (0.28–27.6)	0.384
No (*n* = 102)	53 (52.0%)	49 (48.0%)	1	
Postoperative therapy				
No (*n* = 51)	20 (39.2%)	31 (60.8%)	1	
Radiation alone (*n* = 14)	9 (64.3%)	5 (35.7%)	2.79 (0.82–9.54)	0.102
Chemotherapy alone (*n* = 2)	2 (100%)	0 (0%)	NA	NA
Chemoradiation (*n* = 39)	25 (64.1%)	14 (35.9%)	2.77 (1.17–6.56)	0.021*
Rehabilitation				
Yes (*n* = 106)	56 (52.8%)	50 (47.1%)	NA	
No (*n* = 0)	0	0	NA	NA
Oral care				
Yes (*n* = 105)	55 (52.4%)	50 (47.6%)	NA	
No (*n* = 1)	1 (100%)	0 (0%)	NA	NA
Shape of the tongue				
Protuberant (*n* = 86)	46 (53.5%)	40 (46.5%)	1	
Flat (*n* = 16)	8 (50.0%)	8 (50.0%)	0.87 (0.30–2.53)	0.8
Depressed (*n* = 4)	2 (50.0%)	2 (50.0%)	0.87 (0.12-6.46)	0.891

*ALT* Anterolateral thigh, *ASA* American Society of Anesthesiologists, *CI* Confidence interval, *OR* Odds ratio, *RAM* Rectus abdominis myocutaneous, *TNM* Tumor-node-metastasis

^*^P-values of < 0.05 were considered statistically significant and are indicated with an asterisk.

### Prognostic Factors Associated with Speech Function in Univariate Analysis

All 121 patients who were eligible for analysis underwent tracheotomy at the time of surgery; of these, 119 (98.3%) patients were disconnected from the tracheal tube at the time of assessment. One patient underwent a repeat tracheotomy due to recurrence, and another had a permanent tracheostoma due to aspiration pneumonia. Speech function was categorized as limited (< 7 points on the Hirose scoring system) or non-limited (8–10 points on the Hirose scoring system). Univariate analysis revealed that speech function was negatively affected by age at surgery (*P* = 0.0012), ASA physical status (class 2) (*P* = 0.0004), medical comorbidities of hypertension (*P* = 0.0247) and cardiac dysrhythmia (*P* = 0.0463), primary tumor stage (T4) (*P* = 0.0020), bilateral neck dissection (*P* = 0.0161), laryngeal suspension (*P* = 0.0017), cricopharyngeal myotomy (*P* = 0.0123), and postoperative therapy (radiation only) (*P* = 0.0147). Conversely, the reconstructive procedure of an ALT flap had a positive impact on speech intelligibility (*P* = 0.0415). *P*-values of < 0.05 were considered statistically significant and are marked with an asterisk in Table 3.

**Table 3:** Prognostic factors associated with speech function in univariate analysis (*n* = 121)

Variable	Speech function			
	Limited (%)	Non-limited (%)	OR (95% CI)	*P*
Age at surgery, years				
Median	70	57	1.06 (1.02–1.09)	0.001*
Range	38–83	21–82		
Sex				
Male (*n* = 89)	27 (30.3%)	62 (69.7%)	1	
Female (*n* = 32)	10 (31.25%)	22 (68.75%)	1.04 (0.44–2.50)	0.923
ASA physical status classification				
1 (*n* = 85)	17 (20.0%)	68 (80.0%)	1	
2 (*n* = 33)	18 (54.5%)	15 (45.5%)	4.80 (2.02–11.4)	0.0004*
3 (*n* = 3)	2 (66.7%)	1 (33.3%)	8.00 (0.68–93.5)	0.097
4 (*n* = 0)	0	0	NA	NA
5 (*n* = 0)	0	0	NA	NA
6 (*n* = 0)	0	0	NA	NA
Body mass index, kg/m^2^				
Median	22	21.9	0.99 (0.88–1.12)	0.882
Range	16.9–30.7	15.5–31.6		
Albumin, g/dL				
Median	4	4.2	0.43 (0.16–1.17)	0.099
Range	3.1–5.0	2.9–4.9		
Hemoglobin, g/dL				
Median	13.3	13.7	0.90 (0.70–1.17)	0.434
Range	9.0–16.8	10.4–17.4		
Tobacco use				
Never (*n* = 36)	10 (27.8%)	26 (72.2%)	1	
Ever (*n* = 49)	15 (30.6%)	34 (69.4%)	1.15 (0.44–2.96)	0.777
Current (*n* = 36)	12 (33.3%)	24 (66.7%)	1.30 (0.48–3.56)	0.609
Alcohol consumption				
Never (*n* = 32)	6 (18.75%)	26 (81.25%)	1	
Ever (*n* = 14)	5 (35.7%)	9 (64.3%)	2.41 (0.59–9.84)	0.221
Sometime (*n* = 29)	9 (31.0%)	20 (69.0%)	1.95 (0.60–6.39)	0.27
Almost every day (*n* = 46)	17 (37.0%)	29 (63.0%)	2.54 (0.87–7.41)	0.088
Medical comorbidities				
Hypertension				
Yes (*n* = 44)	19 (43.2%)	25 (56.8%)	2.49 (1.12–5.53)	0.025*
No (*n* = 77)	18 (23.4%)	59 (76.6%)	1	
Diabetes				
Yes (*n* = 19)	3 (15.8%)	16 (84.2%)	1.83 (0.67–5.01)	0.239
No (*n* = 102)	12 (11.8%)	90 (88.2%)	1	
Cardiac dysrhythmia				
Yes (*n* = 10)	6 (60.0%)	4 (40.0%)	3.87 (1.02–14.7)	0.046*
No (*n* = 111)	31 (27.9%)	80 (72.1%)	1	
Peripheral vascular disease				
Yes (*n* = 1)	0 (0%)	1 (100%)	NA	NA
No (*n* = 120)	37 (30.8%)	83 (69.2%)	NA	
Cerebral disease				
Yes (*n* = 7)	4 (57.1%)	3 (42.9%)	3.27 (0.69–15.4)	0.134
No (*n* = 114)	33 (28.9%)	81 (71.1%)	1	
Neuromuscular disorder				
Yes (*n* = 1)	1 (100%)	0 (0%)	NA	NA
No (*n* = 120)	36 (30.0%)	84 (70.0%)	NA	
Psychiatric disorder				
Yes (*n* = 4)	3 (75.0%)	1 (25.0%)	7.32 (0.74–72.9)	0.089
No (*n* = 117)	34 (29.1%)	83 (70.9%)	1	
Preoperative therapy			1	
Radiation				
Yes (*n* = 1)	0 (0%)	1 (100%)	NA	NA
Dose, Gy	–	40		
No (*n* = 120)	37 (30.8%)	83 (69.2%)	NA	
Chemotherapy				
Yes (*n* = 12)	4 (33.3%)	8 (66.7%)	1.15 (0.32–4.09)	0.827
No (*n* = 109)	33 (30.3%)	76 (69.7%)	1	
Surgery				
Yes (*n* = 11)	4 (36.4%)	7 (63.6%)	1.33 (0.37–4.87)	0.663
No (*n* = 110)	33 (30.0%)	77 (70.0%)	1	
TNM classification				
Primary tumor (T)				
T0 (*n* = 0)	0	0	NA	NA
T1 (*n* = 1)	0 (0%)	1 (100%)	NA	NA
T2 (*n* = 9)	1 (11.1%)	8 (88.9%)	0.00 (0.06–4.11)	0.992
T3 (*n* = 67)	14 (20.9%)	53 (79.1%)	1	
T4 (*n* = 42)	21 (50.0%)	21 (50.0%)	3.79 (1.63–8.81)	0.002*
TX (*n* = 0)	0	0	–	NA
Recurrence (*n* = 2)	1 (50.0%)	1 (50.0%)	3.79 (0.22–64.4	0.357
Regional lymph nodes (N)				
N0 (*n* = 56)	12 (21.4%)	44 (78.6%)	1	
N1 (*n* = 16)	6 (37.5%)	10 (62.5%)	2.20 (0.67–7.28)	0.197
N2 (*n* = 41)	16 (39.0%)	25 (61.0%)	2.35 (0.96–5.74)	0.062
N3 (*n* = 6)	2 (33.3%)	4 (66.7%)	1.83 (0.30–11.2)	0.512
NX (*n* = 0)	0	0	NA	NA
Missing (*n* = 2)	1	1	NA	NA
Tongue defect type				
1: (*n* = 55)	11 (20.0%)	44 (80.0%)	1	
2: (*n* = 23)	8 (34.8%)	15 (65.2%)	2.13 (0.72–6.30)	0.17
3: (*n* = 21)	8 (38.1%)	13 (61.9%)	2.46 (0.82–7.40)	0.109
4: (*n* = 16)	7 (43.75%)	9 (56.25%)	3.11 (0.95–10.2)	0.061
5: (*n* = 6)	3 (50.0%)	3 (50.0%)	4.00 (0.71–22.6)	0.117
Resection beyond the tongue				
Yes (*n* = 44)	14 (31.8%)	30 (68.2%)	1.10 (0.49–2.44)	0.823
No (*n* = 77)	23 (29.9%)	54 (70.1%)	1	
Neck dissection				
None (*n* = 0)	0	0	NA	NA
One side (*n* = 46)	8 (17.4%)	38 (82.6%)	1	
Both sides (*n* = 75)	29 (38.7%)	46 (61.3%)	3.00 (1.22–7.31)	0.016*
Reconstructive procedure				
RAM (*n* = 67)	26 (38.8%)	41 (61.2%)	1	
ALT (*n* = 52)	11 (21.2%)	41 (78.8%)	0.42 (0.19–0.97)	0.042*
Forearm (*n* = 1)	0 (0%)	1 (100%)	NA	NA
Groin (*n* = 1)	0 (0%)	1 (100%)	NA	NA
Operation time, min				
Median	508	556	1.00 (1.00–1.00)	0.106
Range	354–1056	385–1250		
Bleeding, mL				
Median	297	345	1.00 (1.00–1.00)	0.986
Range	90–1380	33–1010		
Tracheotomy				
Yes (*n* = 121)	37 (30.6%)	84 (69.4%)	NA	NA
No (*n* = 0)	0	0	NA	
Laryngeal suspension				
Yes (*n* = 49)	23 (46.9%)	26 (53.1%)	3.67 (1.63–8.24)	0.002*
No (*n* = 72)	14 (19.4%)	58 (80.6%)	1	
Cricopharyngeal myotomy				
Yes (*n* = 15)	9 (60.0%)	6 (40.0%)	4.18 (1.36–12.8)	0.012*
No (*n* = 106)	28 (26.4%)	78 (73.6%)	1	
Additional resection				
Yes (*n* = 11)	6 (54.5%)	5 (45.5%)	3.06 (0.87–10.8)	0.081
No (*n* = 110)	31 (28.2%)	79 (71.8%)	1	
Reoperation for flap-related complications				
Yes (*n* = 5)	3 (60.0%)	2 (40.0%)	3.62 (0.58–22.6)	0.169
No (*n* = 116)	34 (29.3%)	82 (70.7%)	1	
Postoperative therapy				
No (*n* = 54)	12 (22.2%)	42 (77.8%)	1	
Radiation alone (*n* = 14)	8 (57.1%)	6 (42.9%)	4.67 (1.35–16.1)	.015*
Chemotherapy alone (*n* = 4)	1 (25.0%)	3 (75.0%)	1.17 (0.11–12.3)	0.898
Chemoradiation (*n* = 49)	16 (32.7%)	33 (67.3%)	1.70 (0.71–4.08)	0.237
Rehabilitation				
Yes (*n* = 121)	37 (30.6%)	84 (69.4%)	NA	
No (*n* = 0)	0	0	NA	NA
Oral care				
Yes (*n* = 120)	36 (30.0%)	84 (70.0%)	NA	
No (*n* = 1)	1 (100%)	0 (0%)	NA	NA
Shape of the tongue				
Protuberant (*n* = 97)	26 (26.8%)	71 (73.2%)	1	
Flat (*n* = 20)	9 (45.0%)	11 (55.0%)	2.23 (0.83–6.01)	0.111
Depressed (*n* = 4)	2 (50.0%)	2 (50.0%)	2.73 (0.37–20.4)	0.327

*ALT* Anterolateral thigh, *ASA* American Society of Anesthesiologists, *CI* Confidence interval, *OR* Odds ratio, *RAM* Rectus abdominis myocutaneous, *TNM* Tumor-node-metastasis

*P-values of < 0.05 were considered statistically significant and are indicated with an asterisk.

## Discussion

The prognosis for resectable OTSCC is relatively good, which raises the need for effective and efficient reconstruction and rehabilitation.^17^ As cancers of the oral cavity and oropharynx can directly affect the function of the teeth, tongue, mandible, palate, and pharynx, patients with OTSCC often present with disruption of their abilities to eat, drink, chew, and swallow.^18^ Beyond our previous retrospective study,^12^ this prospective observational multicenter study focuses on not only the risk of feeding tube dependence but also the food form of oral intake and speech-function-related outcomes for the assessment of patients’ quality of life.

### Prognostic Factors of Postoperative Feeding Tube Dependence in Univariate Analyses

The extent of tongue resection is the most important determinant of functional outcomes after tongue reconstruction.^19–21^ Our data also showed that the extent of tongue defects was directly related to postoperative feeding tube dependence, although there were significant differences in type 3 defects. The lack of a significant outcome for more extensive defects (type 5) may be due to the small number of cases or successful reconstruction with a bulky flap. The RAM flap is used more frequently for extensive tongue reconstruction in Asia than in Western countries, potentially due to differences in body composition across populations. Patients who underwent laryngeal suspension showed a trend toward postoperative feeding tube dependence, although this procedure was applied to improve swallowing function. This is explained by the fact that laryngeal suspension is often performed in patients considered at high risk of dysphagia, including those who have undergone excision of the bilateral suprahyoid muscles.^22^ Surgeons identified these patients as being at high risk of dysphagia and performed the procedure to reduce the risk. However, this did not fully accomplish the goals.

Our results showed that postoperative chemotherapy also increased the risk of postoperative feeding tube dependence, as previously described.^2^ Adjuvant chemotherapy is associated with persistent dysphagia and prolonged gastrostomy tube dependence in patients after transoral robotic surgery.^23,24^ It also increases gastrostomy tube usage and duration of dependency in organ preservation protocols for the treatment of oropharyngeal carcinoma.^25,26^ It is likely that this relationship is mediated by acute cytotoxic effects, including mucositis, gastrointestinal discomfort, dysgeusia, and anorexia, which may develop into chronic maladaptive oral avoidance or aversion.^2^

### Prognostic Factors Associated with Oral Intake Level

The overall rate of feeding tube dependence was only 12.4% (15 patients), which was slightly better than that reported in previous studies;^5,8^ therefore, we focused on the oral intake level among the remaining 87.6% (106) of patients. Advanced age increased the risk of dysphagia after tongue reconstruction, which is consistent with the results reported in previous studies,^4,5^ and was a limiting factor for laryngeal preservation after head and neck reconstruction.^27^ Older patients have less reserve capacity for swallowing than younger patients. Reduced maximal lingual pressure, incomplete relaxation of the upper esophageal sphincter, and delayed triggering of the swallowing reflex are the primary causes of functional loss due to aging.^28,29^ Other prognostic factors of laryngeal suspension and postoperative chemoradiation arise from the same causes as those of postoperative feeding tube dependence.

### Prognostic Factors Associated with Speech Function

Our results showed that younger patients had better speech function according to the Hirose scoring system than older patients, probably because of their reserve capacity and adequate opportunities. A previous study demonstrated that a trend toward worse speech function was observed with advanced age.^4^ Our data also showed that ASA physical status (class 2) and medical comorbidities of hypertension or cardiac dysrhythmia were associated with lower scores, which was expected as these factors are related to advanced age. Primary tumor stage (T4) and bilateral neck dissection were also associated with lower scores in our data, which were expected to be related to tongue defect type, although there was no significant difference, in contradiction to the results of previous studies.^4,30^ This finding suggests that an ALT flap could be used successfully to reconstruct larger defects in our study. A previous study also described that the protuberant shape of the tongue reconstructed using a thicker skin flap, such as a rectus abdominis myocutaneous flap, showed better speech intelligibility after subtotal or total glossectomy for Asian patients.^10,31^ Laryngeal suspension and cricopharyngeal myotomy tended to be applied for larger defects but did not improve conversational function. Our results showed that postoperative radiation was also associated with speech function, as previously described.^4,32^ Radiation therapy to the head and neck causes mucositis, ulceration, and pseudomembrane formation, which are related to oral pain and odynophagia.^18^ In addition, atrophy of the transferred flap due to radiation is inevitable and can adversely affect speech function.^33^

### Limitations

This study has some limitations. Because of the nonrandom treatment assignment, unmeasured confounding variables may have affected surgical outcomes. Given the small number of patients with feeding tube dependence and the subdivision by tongue resection type, the significance of this result should be interpreted with caution. Further multivariate analysis is needed to assess predictive variables, as shown in our previous retrospective study.^12^ Because this was not possible in the current study due to the limited number of events, it will be addressed in a much larger database in the future. Additionally, given the individualized nature of surgical procedures such as laryngeal suspension or cricopharyngeal myotomy and medical regimens, the generalizability of our results may be limited. Furthermore, variations in treatment protocols, such as oral care or rehabilitation, and interobserver differences among surgeons across various institutions over an extended study period are also likely factors.

## Conclusions

The first step in addressing healthcare quality concerns regarding functional outcomes after tongue reconstruction is identifying predictive risk factors. To the best of our knowledge, this is the first prospective observational multicenter study on functional outcomes after tongue reconstruction. We found that extended tongue defect, laryngeal suspension, and postoperative chemotherapy were risk factors for postoperative feeding tube dependence. We also identified some risk factors for swallowing and speech dysfunction. The results of our study complement those of previous studies by providing more robust and detailed clinical data. When planning treatment for high-risk patients, physicians should consider the survival benefits against the functional sequelae of combined modality treatment. These results will allow physicians to set functional expectations more appropriately for reconstructive surgery in OTSCC. The data presented here may also prove useful for postoperative care counseling and managing patient expectations.

## Data Availability

Jun Araki (the principal investigator), Keita Mori (biostatistician), and Yoshichika Yasunaga had full access to all the data analyzed in the study and take responsibility for the integrity of the data and accuracy of the data analysis.
